# Feline thymidine kinase 1: molecular characterization and evaluation of its serum form as a diagnostic biomarker

**DOI:** 10.1186/s12917-021-03030-5

**Published:** 2021-09-27

**Authors:** Liya Wang, Hanan Sharif, Sara Saellström, Henrik Rönnberg, Staffan Eriksson

**Affiliations:** 1 Department of Anatomy, Physiology and Biochemistry, Swedish University of Agricultural Sciences, Uppsala, Sweden; 2 Alertix Veterinary Diagnostic AB, SE-392 30 Kalmar, Sweden; 3 University Animal Hospital, Swedish University of Agricultural Sciences, Uppsala, Sweden; 4 Department of Clinical Science, Swedish University of Agricultural Sciences, Uppsala, Sweden

**Keywords:** Feline thymidine kinase 1, Biomarkers, Feline lymphoma, Inflammatory diseases, Cancer diagnostics

## Abstract

**Background:**

Thymidine kinase 1 (TK1) catalyzes the initial phosphorylation of thymidine in the salvage pathway synthesis of dTTP, an essential building block of DNA. TK1 is a cytosolic enzyme with its highest level during the S-phase of the cell cycle. In cancer cells TK1 is upregulated and excess TK1 is leaked into the blood. Therefore, serum TK1 has been used as biomarker for cancer diagnosis and prognosis in human medicine. Feline TK1 shows high sequence similarity to TK1 from other species. The aim of this study was to characterize feline TK1 and evaluate if serum TK1 can be used as a diagnostic biomarker.

**Results:**

Feline TK1 was cloned, expressed and affinity purified. The purified feline TK1 phosphorylated not only pyrimidine deoxyribonucleosides but also pyrimidine ribonucleosides and to some extent purine deoxynucleosides. A number of anticancer and antiviral nucleoside analogs also served as substrates with fairly high efficiency. ATP and dATP were the preferred phosphate donor. Serum TK1 activity in felines with malignant diseases was significantly higher than that in healthy individuals. ROC analysis revealed an area under the curve (AUC) of 0.98 with a sensitivity of 0.83 and a specificity of 0.95 for felines with lymphoma. Serum TK1 activity in felines with IBD or inflammatory disease was within the same range as healthy ones. Furthermore, in felines with lymphoma serum TK1 activity returned to normal levels in response to treatment.

**Conclusion:**

Feline TK1 has high specific activity and a broader substrate specificity in comparison with TK1 from other species. Serum TK1 activity in felines with malignant diseases is significantly higher than that in normal felines and in felines with inflammatory diseases. These results suggest that serum TK1 may be a promising biomarker for the diagnosis and monitoring of malignant diseases and for the differential diagnosis of certain inflammatory disease.

**Supplementary Information:**

The online version contains supplementary material available at 10.1186/s12917-021-03030-5.

## Background

Thymidine kinase 1 (TK1) catalyzes the transfer of a gamma phosphate group from ATP to the 5′-OH group of thymidine (dThd), leading to the formation of thymidine 5′-monophosphate, which is further phosphorylated to thymidine triphosphate (dTTP), an essential building block of DNA. The expression of TK1 is cell cycle regulated and coincides with DNA replication. The level of TK1 is highest during the S-phase of the cell cycle and it drastically declines during mitosis [1, 2], and this is achieved by the ubiquitin-proteasome regulated pathway [3]. In cancer cells, TK1 is upregulated and the usual cell cycle regulated degradation during mitosis is disrupted and excess TK1 leaks out and it can be detected in serum from patients with malignant diseases and also in some cases of bacterial infection and/or inflammatory diseases [4, 5]. In human medicine, serum TK1 has been used in health screening and for cancer diagnosis and prognosis [6, 7]. In veterinary medicine, serum TK1 have been evaluated as biomarker for diagnosis and prognosis primarily in canines with hematological malignancies [8–10]. However, feline TK1 has not been characterized, although it is expected that it should have similar properties to TK1s from other mammalian species.

The prevalence of cancer increases with age in felines, and approximately 32% of felines ≥10 years old will eventually develop cancer. Lymphoma is the most common neoplastic disease in felines, accounting for approximately 30% of all cancer cases. Among the gastrointestinal malignancies, up to 55% are alimentary lymphoma [11, 12], which is characterized by neoplastic lymphocyte infiltration of the gastrointestinal tract and/or associated lymph nodes. Clinical signs include weight loss, vomiting, diarrhea, and changes in appetite. A definitive diagnosis of cat lymphoma often requires histopathological examination of full-thickness gastrointestinal biopsies. In cases of low grade alimentary lymphoma histological assessment of biopsies is required, whereas intermediate and high grade alimentary lymphoma can be diagnosed by cytology of intestinal or mesenteric lymph node aspirates using fine needle aspiration [11, 12].

Inflammatory bowel disease (IBD), a common gastrointestinal disorder in elderly felines, comprises a heterogeneous group of immunologically mediated disorders of the gastrointestinal tract. One of the characteristics of IBD is inflammatory cell infiltration of the mucosal layer, such as T-lymphocytes, plasma cells and also eosinophils, neutrophils and macrophages. The clinical signs of IBD are similar to those of gastrointestinal lymphoma. Therefore, the diagnosis of IBD is complicated, which requires careful exclusion of IBD mimics. Fecal analysis, serum biochemistry, radiography, ultrasonography, as well as histopathological analysis of biopsies are necessary to reach a correct IBD diagnosis [11, 13].

The treatment and the prognosis of IBD and gastrointestinal lymphoma differ significantly, and therefore, it is vital to make the correct diagnosis. However, it is usually not possible to distinguish low grade alimentary lymphoma from other feline gastrointestinal diseases such as IBD or benign lymphoid hyperplasia since these diseases share similar clinical signs. Differentiating IBD from gastrointestinal lymphoma in felines is a diagnostic challenge since physical examination, ultrasonography and gross examination (e.g. both endoscopic and surgical visualization) are of limited use. The potential of IBD to progress to lymphoma further complicates the diagnosis [11–14]. Therefore, less invasive methods that can differentiate the two diseases are highly desirable.

Serum biomarkers are valuable and cost effective tools for in vitro diagnostics. The development of serum biomarkers that can differentiate alimentary lymphoma from IBD has thus a high priority. Lactate dehydrogenase (LDH) is a well-established serum biomarker in humans; it is elevated in sera from patients with various cancer diseases, and the levels of serum LDH are inversely correlated to patient survival [15]. However, recent studies have shown that serum LDH has limited value in differentiating cancer from healthy and non-neoplastic diseases in canines [16], as well as in differentiating alimentary lymphoma from IBD in felines [17].

The aim of this study was to conduct a biochemical characterization of feline TK1 and to evaluate if serum TK1 can be used as a biomarker in clinical diagnostics. Feline TK1 cDNA was cloned into the pET-14b vector and expressed in *E. coli*, and the recombinant feline TK1 was affinity purified and characterized. Serum samples from healthy and diseased felines were collected and the levels of TK1 were measured.

## Results

### Molecular characterization of feline TK1

#### Feline TK1 sequence analysis

**Fig. 1:**
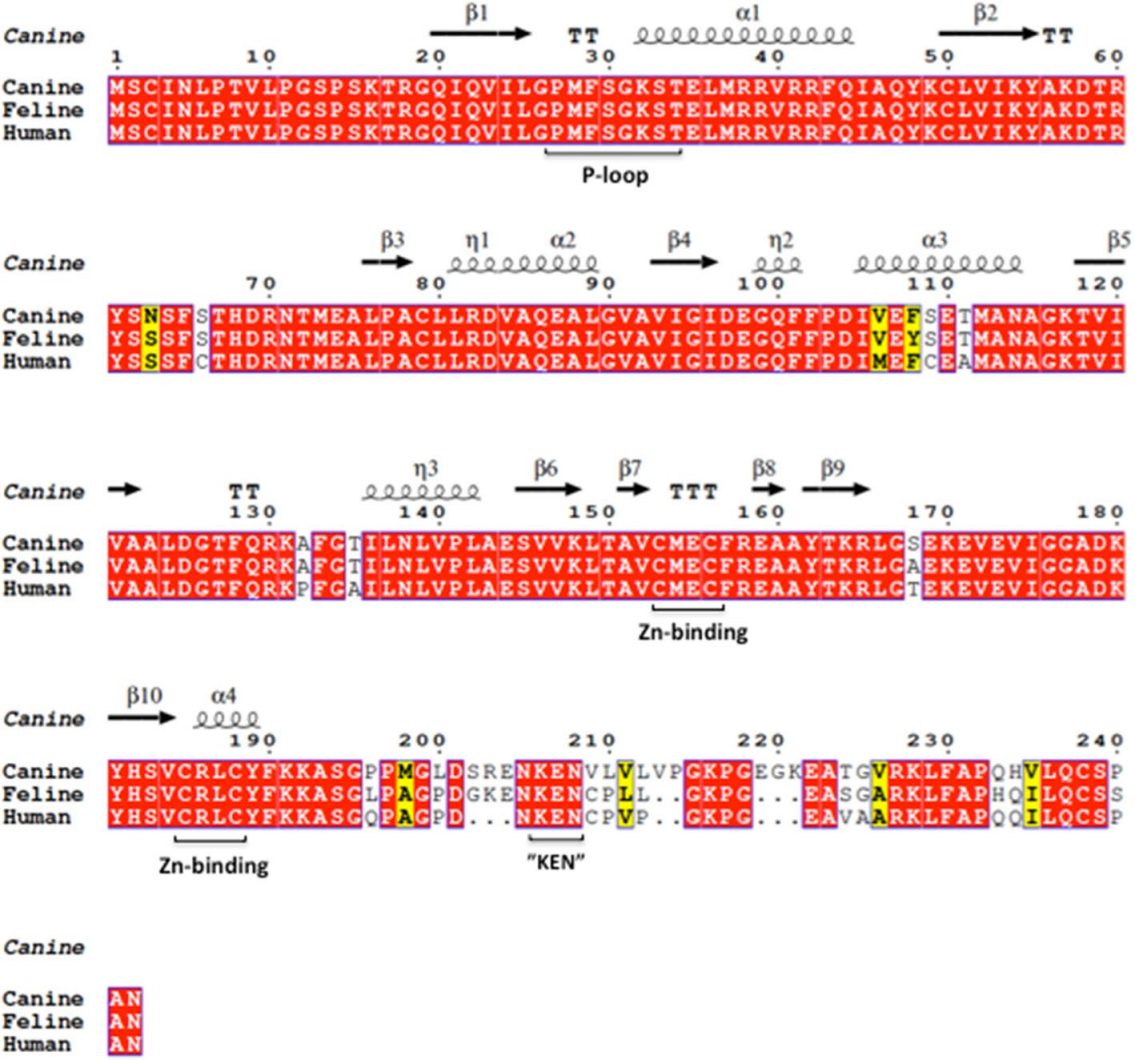
Amino acid sequence alignment of TK1 from feline, canine and human using the Clustal Omega algorithm (https://www.ebi.ac.uk/Tools/msa/clustalo/) and structural alignment by using the ENDscript 3.0 software (http://endscript.ibcp.fr/ESPript/cgi-bin/ENDscript.cgi) with the human TK1 structure as the template [18]

The feline TK1 gene (*tk1*) is 10,729 bp long and it is located on chromosome E1 and has 7 exons. The open reading frame of feline TK1 cDNA encodes a polypeptide of 237 amino acids with a calculated molecular weight of 27.94 kDa. The TK1 sequences are highly conserved among different species, and at the amino acid level, feline TK1 has > 95% sequence identity to canine and human TK1 (Fig. 1). Structural alignment of feline, canine and human TK1 shows that important functional motifs such as the ATP binding “p-loop”, the two “–CXXC- “Zn-binding motifs that form the “Lasso” loop, and the “KEN” motif that regulates proteolytic degradation of TK1 after mitosis, are all identical [18, 19]. The only region that shows sequence diversity is located at the C-terminus (Fig. 1). The C-terminal region of TK1 is essential for mitotic degradation of the enzyme in vivo [3]. In vitro, deletion of the C-terminal 44 amino acids of human TK1 results in a 6-fold increase in its half-life and a 4-fold decrease in k_cat_ value as compared with the wild type enzyme [20]. Thus, the C-terminal sequence plays an important regulatory role in TK1 stability and catalysis. However, the structure of the C-terminal sequence has not been determined experimentally, probably because this region is highly flexible in structure and is surface exposed. This property has been successfully used to design antigens to generate specific antibodies [21, 22].

### Substrate specificity

**Fig. 2:**
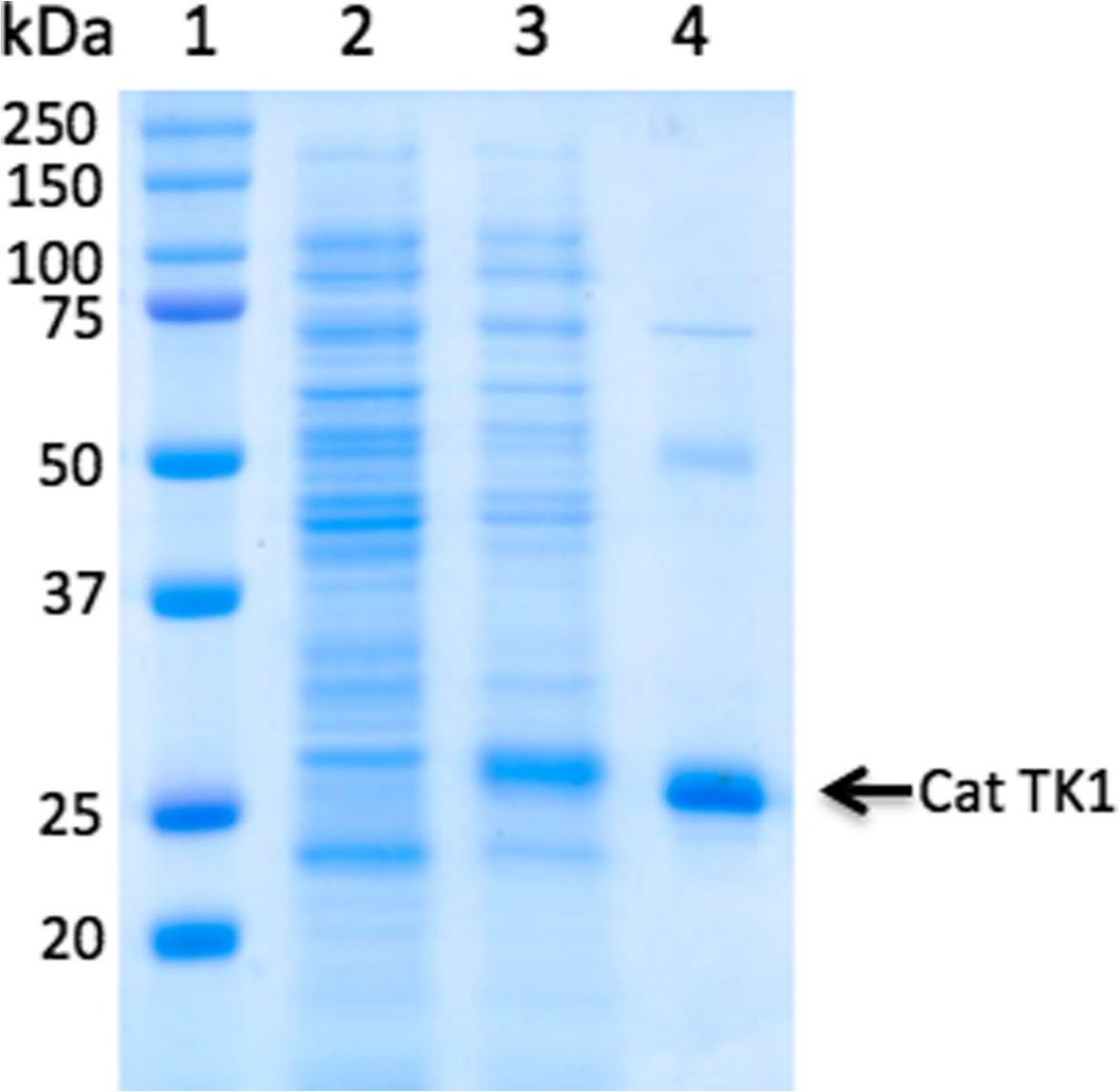
SDS-PAGE analysis of recombinant feline TK1. Lane 1, protein ladder; Lane 2, total extracts of uninduced culture; Lane 3, total extracts of the induced culture; and Lane 4, purified feline TK1

In order to characterize the biochemical and enzymatic properties of the feline enzyme, feline TK1 cDNA was cloned into the pET-14b expression vector and the recombinant feline TK1 protein was expressed in *E. coli* as a fusion protein with an N-terminal 6 x histidine tag in order to facilitate purification. The recombinant feline TK1 protein was then affinity purified to more than 95% purity by using Ni^2+^-Sepharose column chromatography (Fig. 2). The yield of the pure feline TK1 was ~ 5 mg per liter culture.

In the TK1 catalyzed reaction, a gamma phosphate group from a nucleoside triphosphate (phosphate donor) is transferred to the 5′-OH group of a nucleoside (phosphate acceptor), resulting in the formation of nucleoside 5′-monophosphate. Therefore, we studied both phosphate acceptor and phosphate donor specificity. Using ATP as the phosphate donor, we tested both pyrimidine and purine nucleosides and their analogs using a coupled spectrophotometric method. As shown in Table 1, at the 100 μM concentration, the activities of all other substrates were compared with that of thymidine (dThd) (set to 100%); dThd analogs TFT, AZT, FLT, α-dThd and D4T showed lower relative activities than that of dThd. Surprisingly, deoxyuridine (dUrd) and its analogs 5-fluoro-2′-deoxyuridine (5FdU) showed higher relative activity than that of dThd, but other tested dUrd analogs, e.g., L- and D-FMAU, FMAU, and BvDU, showed low relative activity. Uridine (Urd) and deoxyguanosine (dGuo) could also serve as substrates, but not dCyd, cytidine and dAdo, under these conditions (Table 1).

**Table 1:** Substrate specificity

Substrate	Relative activity
dThd	100
Triflurothymidine (TFT)	80.0
Azidothymidine (AZT)	51.8
2′-deoxy-3′-fluorothymidine (FLT)	33.5
α-dThd	5.1
Dideoxythymidine (D4T)	4.1
dUrd	146
5-fluorodeoxyuridine (5FdU)	122
2′-deoxy-2′-fluoro-arabinofuranosyl-5-methyluracil (FMAU)	47.2
D-FMAU	65.5
L-FMAU	20.4
5-Bromovinyl-deoxyuridine (BvdU)	6.8
Uridine	0.9
dCyd	< 0.01
dAdo	< 0.01
dGuo	0.2

The assays were performed by using a coupled spectrophotometric method at 21 °C. The concentration of the substrate was 0.1 mM with 1 mM ATP as the phosphate donor. The data are given as percentage of that with dThd as the substrate (1.64 μmol/mg/min)

Using 100 μM [^3^H]-dThd as the phosphate acceptor, we studied the phosphate donor specificity using radiochemical assays. Except for dTTP, all tested nucleoside triphosphates at a 1 mM concentration could serve as phosphate donors. ATP and dATP showed the highest activities and other nucleoside triphosphates had 12–18% activities as compared with ATP (Table 2). These results showed that the feline enzyme has a preference for adenine as the base of the phosphate donor but there is no restriction on the sugar moiety since both ATP and dATP have similar activities and are the best phosphate donors. dTTP, the end product of dThd phosphorylation, could not act as phosphate donor (Table 2) but most likely it acts as a feed-back inhibitor. Thus, feline TK1 shows similar substrate requirements to human TK1 but with a broader substrate specificity [23].

**Table 2:** Phosphate donor specificity

Phosphate donor	Relative activity
ATP	100
GTP	16.4
CTP	13.1
UTP	18.9
dATP	97.1
dGTP	10.8
dCTP	12.6
dTTP	< 0.01

The assays were performed by using a radiochemical assay with [^3^H]-dThd as the substrate at 37 °C. The concentration of dThd was 0.1 mM and the phosphate donor was 1 mM. The data are given as percentage of that with ATP as the phosphate donor (2.56 μmol/mg/min)

### Steady-state kinetics

**Fig. 3:**
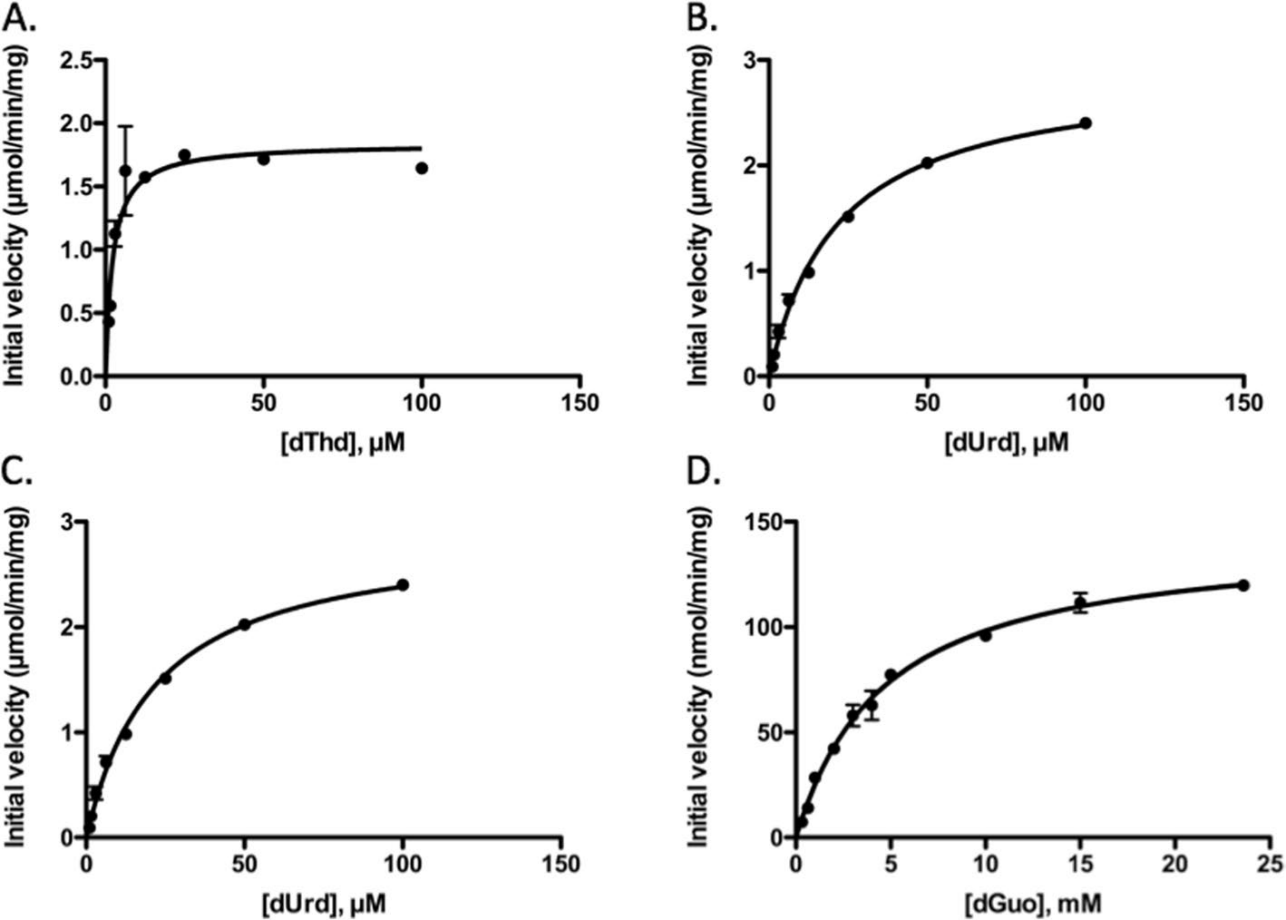
Steady-state kinetic analysis of feline TK1 with the phosphate acceptor as the variable substrate. Plots of the initial velocity versus the substrate concentration, dThd (**A**), dUrd (**B**), Urd (**C**) and dGuo (**D**). The ATP concentration was kept at 1 mM

Steady-state kinetic analysis was conducted by using a coupled spectrophotometric method. The phosphorylation of dThd showed positive cooperativity with a Hill coefficient of 1.9 (Fig. 3A). The kinetic parameters K_M_ value was 2.25 μM and the V_max_ value was 1.84 μmol/min/mg for dThd (Table 3). The phosphorylation of dUrd, however, followed Michaelis-Menten kinetics (Fig. 3B) with K_M_ and V_max_ values of 22.0 μM and 2.91 μmol/min/mg, respectively. Due to the high K_M_ value, the efficiency for dUrd phosphorylation was only 16.1% of that of dThd (Table 3).

**Table 3:** Kinetic parameters

Substrate	K_M_ (μM)	V_max_ (μmol/min/mg)	Efficiency (V_max_/K_M_)	Hill coefficient (n)
dThd	2.25 ± 0.49	1.84 ± 0.09	0.82 (100)*	1.9
dUrd	22.0 ± 1.57	2.91 ± 0.08	0.13 (16.1)	0.9
5FdU	3.13 ± 0.52	2.02 ± 0.08	0.64 (78.8)	0.6
AZT	1.60 ± 0.28	0.94 ± 0.03	0.59 (72.0)	2.1
TFT	3.54 ± 0.28	1.41 ± 0.02	0.40 (48.7)	1.4
Urd	4850 ± 300	0.53 ± 0.01	0.00011 (0.013)	1.0
dGuo	4690 ± 372	0.14 ± 0.004	0.000031 (0.0038)	1.0
ATP	256 ± 37	1.94 ± 0.14	0.0030	2.2

Kinetic parameters were determined by using a coupled spectrophotometric method at 21 °C. To determine the kinetic parameters for the phosphate acceptors, the ATP concentration was fixed at 1 mM and to determine the kinetic parameters for ATP, the dThd concentration was fixed at 100 μM. The K_M_ and V_max_ values were determined by fitting the initial velocity data into the Michaelis-Menten equation. The Hill coefficient (n) was calculated by fitting the initial velocity data into the Hill equation

The kinetic parameters of the selected nucleoside analogs that are used in antiviral and anticancer therapy were also determined. The phosphorylation of 5FdU showed negative cooperativity while trifluorothymidine (TFT) and AZT demonstrated positive cooperativity (Table 3). The K_M_ values for these analogs were 3.13 (5FdU) μM, 3.54 (TFT) μM, and 1.60 (AZT) μM, which were similar to that of dThd, but the V_max_ values varied, and thus the efficiency for these analogs were lower than that of dThd (Table 3).

Since Urd and dGuo also showed activity in the initial tests, their kinetic parameters were also determined. As shown in Fig. 3C and D the phosphorylation of Urd and dGuo followed Michaelis-Menten kinetics with K_M_ values of 4850 and 4690 μM, and V_max_ values of 0.53 and 0.14 μmol/min/mg, respectively (Table 3).

**Fig. 4:**
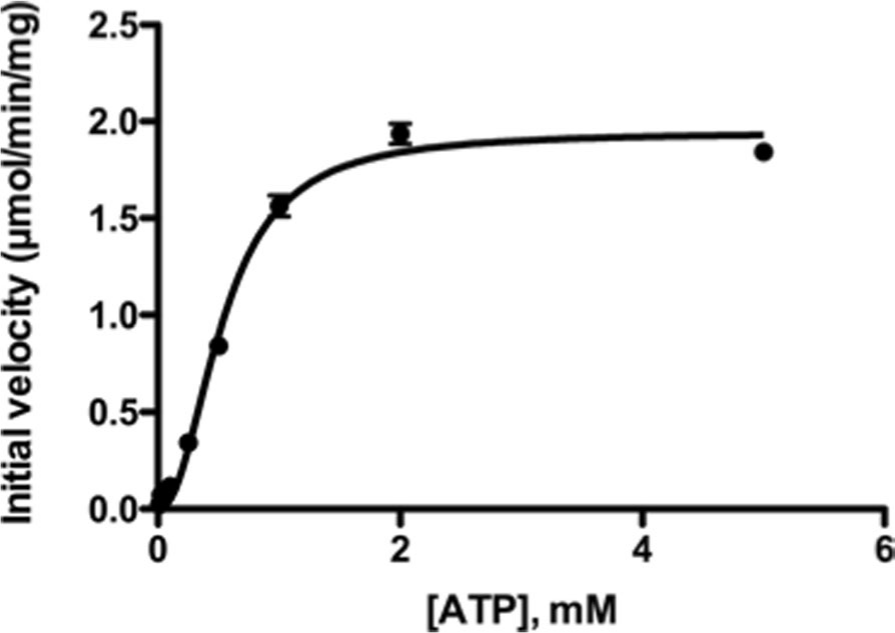
Steady-state kinetic analysis of feline TK1 with ATP as the variable substrate. The dThd concentration was kept at 0.1 mM

With ATP as the variable substrate and a fixed dThd concentration, the ATP saturation showed positive cooperativity with a Hill coefficient of 2.2 (Fig. 4). The K_M_ and V_max_ values for ATP were 794 μM and 2.38 μmol/min/mg respectively (Table 3). These results suggest that the binding of ATP may affect subunit interactions as seen in cases of human TK1, i.e., at higher ATP concentrations the enzyme is in a tetramer form with higher catalytic activity, a typical behavior of a cooperative or allosteric enzyme [24].

Taken together, cat TK1 phosphorylated its presumably natural substrate dThd with the highest efficiency, and the enzyme exhibited broader substrate specificity and higher catalytic rate as compared with TK1 from humans and canines [23, 25]. ATP as the best phosphate donor may also act as a type of allosteric regulator.

### Serum sample collection and serum TK1 analysis

Serum samples from healthy and diseased felines were collected; a total of 40 serum samples were collected from healthy felines, and 49 sera were collected from diseased ones, including malignant lymphoma (naïve), lymphoma before and after treatment, mastocytoma, brain tumor, and inflammatory diseases such as IBD.

TK1 activity in serum samples was determined by using [^3^H]-dThd and ATP as substrates, a method developed previously for canine serum TK1 determination [26] but also shown here to be optimal for feline serum TK1 activity measurement. The mean serum TK1 activity in normal serum samples (healthy felines) was 0.53 pmol/min/ml with a standard deviation (SD) of 0.22 pmol/min/ml, and thus we used a cutoff value of 0.97 pmol/min/ml (i.e., mean + 2xSD).

**Fig. 5:**
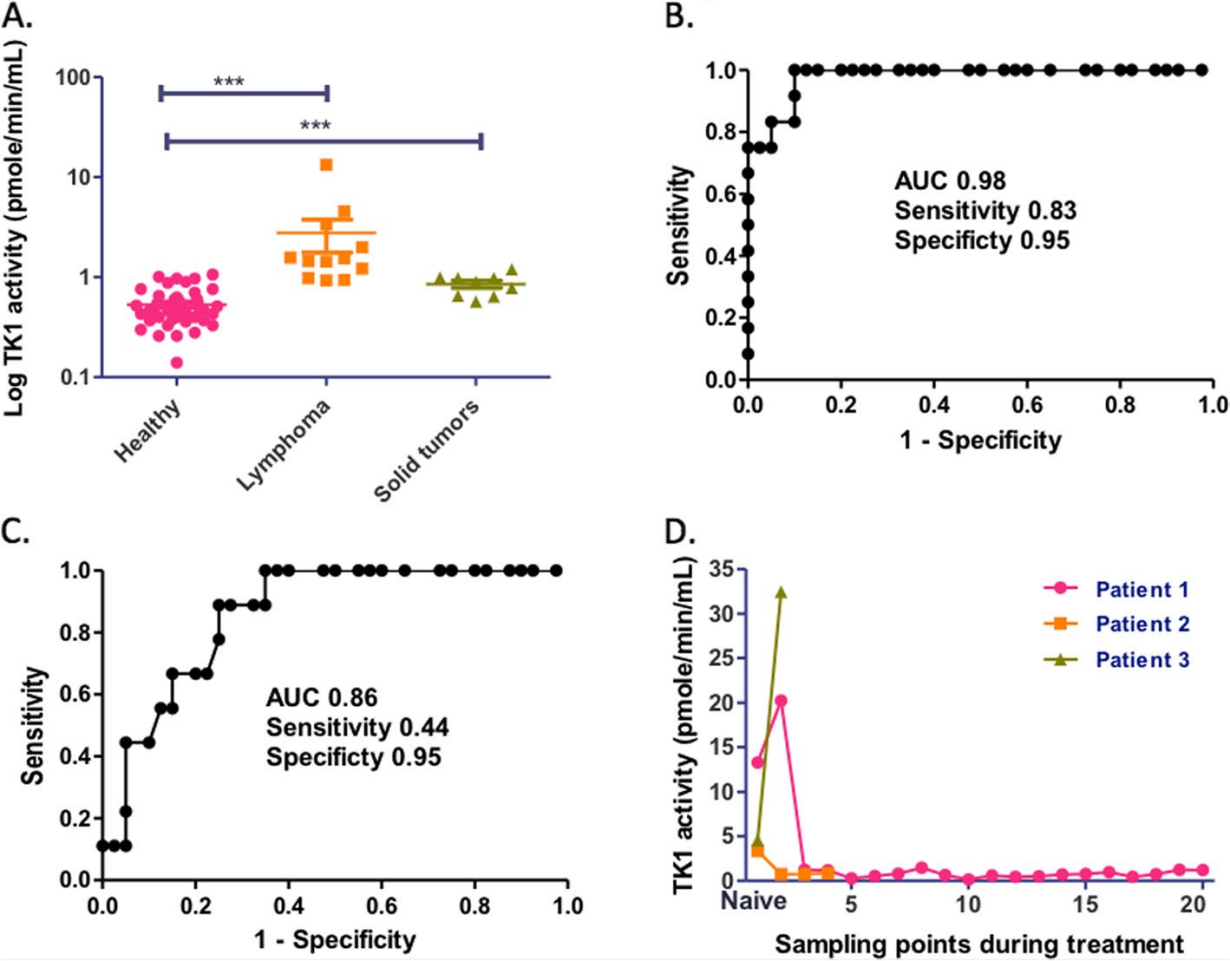
Analysis of serum TK1 levels in normal and diseased cats. **A** Comparison of serum TK1 levels in healthy group (*n* = 40) and cats with malignant diseases (lymphoma *n* = 12, and solid tumor *n* = 9); **B** ROC (receiver operating characteristic) analysis of serum TK1 levels in lymphoma group; **C** ROC analysis of serum TK1 levels in the solid tumor group; **D** Serum TK1 levels in 3 patients following treatment. The sampling point corresponded to the time when the serum sample was taken during a veterinary visit and a new treatment dose was given if needed. The time interval between each sampling point varied from 1 to 3 weeks

Serum TK1 activity in samples from felines with malignant lymphoma ranged from 1.5 to 13.3 pmol/min/ml, all of which were clearly above the cutoff value. As shown in Fig. 5, serum TK1 activities in felines with malignant diseases were significantly higher than those in healthy individuals. ROC (receiver operating characteristic) analysis showed an AUC (area under the curve) value of 0.98 (*p* <  0.0001) for the lymphoma group and 0.86 (*p* <  0.001) for the solid tumor group, at 95% confidence interval. At the chosen cutoff value the sensitivity was 0.83 and the specificity was 0.95 for lymphoma (Fig. 5B) and for the solid tumor group, the values were 0.44 and 0.95, respectively (Fig. 5C). Thus, the sensitivity of the present TK1 activity assay is much improved compared to what was reported in an earlier study using the TK-REA assay where the ROC analysis showed an AUC value of 0.66, a sensitivity value of 0.40 and a specificity value of 0.94 [5]. Our results suggest that serum TK1 activity may serve as a useful biomarker to distinguish felines with malignant diseases from healthy ones.

We have followed three patients with lymphoma who had undergone chemotherapy by monitoring their serum TK1 activity before and after treatment and at later follow-ups. As shown in Fig. 5D, before the therapy started, the serum TK1 activities of these patients ranged from 1.50 to 13.3 pmol/min/ml, which were clearly above the cutoff value. After initial treatment, two of the patients’ serum TK1 activities were decreased to 0.17 to 1.47 pmol/min/ml, respectively, at the third sampling point, which were significantly lower than those before treatment. During the follow-up veterinary visits (the time between each sampling point/veterinary visit varied from 1 to 3 weeks), their serum TK1 activities remained low, within the same range as those of healthy individuals (Fig. 5D). These two patients were in complete remission. The third patient did not respond to vincristine treatment and 1 week later the patient’s clinical condition deteriorated, and the serum TK1 activity also increased significantly. This patient suffered from a large granular cell lymphoma, which is a very progressive and aggressive form of lymphoma, and the patient had to be euthanized. Thus, the levels of serum TK1 activity correlated with the clinical disease progression during therapy. These results suggest that serum TK1 activity can be used to monitor the treatment of felines with malignant lymphoma, as has been shown in canines [26].

Serum samples collected from felines with confirmed IBD or inflammatory disease (*n* = 2) but not lymphoma had serum TK1 activity ≤0.5 pmol/min/ml, which is within the normal range (Supplementary Table 1).

## Discussion

In this study we have cloned, expressed and characterized the feline TK1. Our results show that the feline TK1 has a broader substrate specificity and a preference both for the nucleobases and sugar moiety of its substrates. On the nucleobase level, the feline TK1 preferred thymine and uracil over cytosine, since only at a very high concentration did dCyd show any detectable activity. Among the tested purine nucleosides, dGuo showed some activity at 0.1 mM but dAdo showed no activity even at > 10 mM concentration (Table 1). Modifications of the nucleobase, e.g. BvDU, resulted in reduced activity (Table 1). In cases of the sugar moiety, feline TK1 prefers deoxyribose over ribose and this could be seen with dUrd and its corresponding ribonucleoside Urd; Urd gave only 0.9% relative activity while dUrd had 146% relative activity as compared with that of dThd. Thymidine analogs with modifications on the sugar moiety such as AZT, FLT, and D4T all had reduced activity (Table 1).

The α-configuration is an anomer of the β-configuration and is very rare in nature. With feline TK1, the α-dThd had 5% activity as compared with the β-configuration. Except for the α- and β-configuration, the sugar moiety can also be in either L- or D- enantiomeric forms. We tested D- and L- configurations and a racemic mixture of FMAU, and found that D-FMAU had 3 times the activity of L-FMAU and the racemic mixture of FMAU had activity that was within the average of the D- and L-forms (Table 1). These results are similar to those of human TK1 [27].

Steady-state kinetic analysis revealed that some substrates followed Michaelis-Menten kinetics but others did not, and their kinetics followed the Hill equation with Hill coefficient < 1 or > 1, i.e., either positive cooperative or negative cooperative, demonstrating a possible cooperative or allosteric behavior of feline TK1 toward certain substrates (Table 3). For example, the phosphorylation of AZT showed positive cooperativity with a Hill constant of 2.1, which is similar to canine TK1. However, with human native TK1, the phosphorylation of AZT followed the Michaelis-Menten kinetics [23, 25]. The ATP kinetics showed positive cooperativity, suggesting that ATP binding affected subunit interactions, similar to human TK1, which showed a concentration dependent transition between the tetramer form with a higher catalytic activity and the dimer form with a lower catalytic activity [24].

Nucleoside analogs have been widely used in the treatment of viral infections and various cancers, and today more than 50% of antiviral and anticancer drugs are nucleobases, nucleoside and nucleotide analogs. Our results showed that the anticancer drug 5FdU and the antiviral and anticancer drug TFT had > 70% efficiency, and the anti-HIV drug AZT had ~ 49% efficiency of that of dThd. L-FMAU, an anti-hepatitis B viral drug, was also phosphorylated at a relative high activity, similar to human TK1 [28]. Nucleoside analogs have been used in cancer treatment for canines and are well tolerated. Our results demonstrated that feline TK1 can effectively phosphorylate antiviral and anticancer nucleoside analogs, which may suggest a future use of nucleoside analogs in the treatment of feline cancers.

Serum TK1 has been used as a biomarker in health screening, cancer diagnosis and prognosis in human medicine [7, 29]. In veterinary medicine, canine serum TK1 activity has also been shown to be a useful diagnostic and prognostic biomarker for hematological malignancies and it can be used to monitor cancer treatment [8–10, 25, 30]. However, for canines with solid tumors serum TK1 activity showed no significant difference from healthy individuals, and therefore, an ELISA method that measures serum TK1 protein levels has been developed. It has been shown that serum TK1 protein levels in patients with solid tumors are significantly higher than those of healthy individuals. Thus, it may be suitable for diagnosis and prognosis [8, 22, 26, 30].

In this study, we evaluated if feline serum TK1 could be used for discriminating cancer from other diseases and showed that felines with malignant diseases had significantly higher serum TK1 activity than those of healthy ones. Our ROC analysis revealed an AUC value of 0.98, a sensitivity value of 0.83 and specificity value of 0.95 for lymphoma group, suggesting that serum TK1 may be a promising biomarker for lymphoma diagnostics and treatment monitoring. Although the sensitivity and specificity for the solid tumor group were not as good as the lymphoma group, the overall serum TK1 activity in this group was significantly higher than that of the healthy group. Therefore, future development of an immunological detection method such as ELISA to measure feline serum TK1 protein levels is highly warranted.

As described earlier, cancer prevalence is increasing with age in felines and lymphoma is the major cancer form in elderly felines. Diagnosis of alimentary lymphoma is often complicated by other gastrointestinal diseases such as IBD, a common gastrointestinal disorder in elderly felines. We showed here that serum TK1 activity in felines with IBD or inflammatory disease was in the same range as normal individuals, albeit a small number of samples were included in this study. Further studies with large sample numbers from IBD patients will help to determine the usefulness of serum TK1 in the differential diagnosis of IBD and intestinal lymphoma.

## Conclusion

The feline TK1 has been purified and characterized and showed broader substrate specificity as compared with TK1 from other species, phosphorylating not only pyrimidine deoxyribonucleosides but also pyrimidine ribonucleoside and to some extent purine deoxynucleosides. A number of anticancer and antiviral nucleoside analogs were also phosphorylated with reasonably high efficiency. Regarding the phosphate donor specificity, feline TK1 preferred ATP or dATP as the phosphate donor. This information can serve as a starting-point for further studies of the basic molecular biology of feline TK1 as well as its use in diagnostics and therapeutics of feline malignant and inflammatory diseases.

Serum TK1 activity in felines with malignant diseases is significantly higher than those of healthy ones. We also observed that serum TK1 activity in felines with IBD or inflammatory disease was within the range of normal healthy ones, and serum TK1 activity returned to normal levels in response to treatment in felines with lymphoma. Although the number of samples was small in this study, our results suggest that feline serum TK1 is a promising biomarker for feline cancer diagnosis and prognosis, for monitoring treatment of malignant diseases, and it may also be used to differentiate IBD from alimentary lymphoma, although future studies with more samples are necessary. The results presented here can serve as a necessary start point for the development of new and more efficient immunodiagnostic tools for diagnostic and prognostic purposes.

## Methods

### Expression and purification of feline TK1

The feline TK1 gene was identified in the database and the cDNA coding for the feline TK1 protein was synthesized and cloned into the pET-14b vector (Genscript Biotech). The recombinant feline TK1 contains a 6 x Histidine tag and a thrombin cleavage site that is fused to the N-terminal of the feline TK1 sequence. Plasmid DNA containing the feline TK1 cDNA was transformed into the *E. coli* BL21 (DE3) pLysS strain, which was cultured in LB medium containing the appropriate antibiotics. The expression of the recombinant protein was induced by the addition of 0.1 mM IPTG to the culture medium and it was purified essentially as previously described [31]. Glycerol (10%) and dithiothreitol (DTT, 5 mM) were added to the feline TK1 preparation and stored at − 70 °C in aliquots until further analysis. The purified feline TK1 was analyzed by SDS-PAGE and the protein concentration was determined by using a Bio-Rad protein assay with bovine serum albumin as the standard.

### TK1 activity determination and steady-state kinetics

The stock solutions of all nucleosides and nucleoside analogs were prepared in DMSO and diluted in distilled water before use. The highest DMSO concentration in the reaction was 10%, which did not interfere with the TK1 activity measurement when tested with dThd as the substrate.

The initial velocities of the feline TK1 catalyzed reaction with different substrates were determined by using a coupled spectrophotometric method [32, 33]. Briefly, the reaction mixture contained 10 mM Tris/HCl, pH 7.6, 5 mM DTT, 5 mM MgCl_2_, 0.5 mM phosphoenolpyruvate, 0.1 mM NADH, 4 unit/ml pyruvate kinase, 4 unit/ml lactate dehydrogenase, 1 mM ATP, and variable concentrations of different substrates in a total volume of 0.5 ml. The reaction was started by the addition of purified feline TK1 (10 μg/ml) and the reduction of NADH concentration was monitored at 340 nm over time. The rate of the product formation was equal to the rate of NADH oxidation in the reaction mixture. All assays were repeated 3–6 times and the results are given as mean ± SD. The kinetic parameters were calculated by fitting the initial velocity data to the Michaelis-Menten equation, V_0_ = V_max_ [S]/(K_M_+[S]) or the Hill equation, V_0_ = V_max_ [S]^n^/(S_1/2_ + [S]^n^), n = Hill coefficient, S_1/2_ is the substrate concentration required to reach ½ V_max_.

### Serum sample collection

Serum samples from healthy felines (*n* = 40) and felines with different malignant diseases (lymphoma group *n* = 38, solid tumors *n* = 9) and inflammatory diseases (*n* = 2) were collected at the University Animal Hospital, Department of Clinical Sciences, Swedish University of Agricultural Sciences, Uppsala, Sweden (see Supplementary Table 1 for details). The samples were stored at − 20 °C until analysis. This project was approved by the Swedish Animal Protection Ethical Committee (ref no. C12/15) and the samples were collected from patients with signed informed consent provided by their owner. The felines were sampled on their first visit at the oncology service and thereafter at revisits, mostly determined by the chemotherapy administration protocol. All experiments were conducted in accordance with ARRIVE guidelines (https://arriveguidelines.org) and regulations presented by the Swedish Animal Protection Ethical Committee.

### Serum TK1 activity measurement

TK1 activity in serum samples was determined by using [^3^H]-dThd (PerkinElmer) as the substrate essentially as previously described [26]. The reaction mixture, containing 10 mM Tris–HCl pH 7.6, 2 mM DTT, 5 mM MgCl_2_, 5 mM NaF, 5 mM ATP, 5 μM [^3^H]-dThd, 10 mM NH_4_Cl and 10 μl serum without dilution in a total volume of 40 μL, was incubated at 37 °C for 60 min. Aliquots of the reaction mixture were spotted onto DEAE filter paper (DEAE filtermat, PerkinElmer) and dried. The filters were then washed 2 times in 1 mM ammonium formate. Thereafter, the filters were sorted into vials, the products were eluted with 0.5 ml buffer (0.1 M HCl and 0.2 M KCl) and counted in a scintillation counter (Tri-Carb, PerkinElmer) after the addition of scintillation fluid (Optisafe, PerkinElmer). All samples were assayed at least 3 times and the results are given as mean ± SD.

### Statistical analysis

Distributions of the TK1 activity levels in the healthy and diseased groups were evaluated for normality using the D’Agostino and Pearson omnibus normality test. Serum TK1 activity levels showed non-Gaussian distributions, and the Mann-Whitney U test were used for the comparisons between groups. Receiver operating characteristic (ROC) curves were constructed in order to evaluate the performance of the feline TK1 activity assay. All statistical analyses were performed using GraphPad Prism 5.0 (GraphPad Software, La Jolla, CA, USA). The ROC curves and the median survival were calculated with the Kaplan-Meier curve using MedCalc version 17.6 (MedCalc software, Ostend, Belgium) statistical discovery software. A *P*-value < 0.05 was considered significant.

## Supplementary Information

**Additional file 1.**

## Data Availability

The authors confirm that all of the raw data supporting the findings of this study are either presented in the article or can be found in the supplementary materials.

## Abbreviations

TK1: Thymidine kinase 1
dThd: 2′-deoxythymidine
dUrd: 2′-deoxyuridine
dCyd: 2′-deoxycytidine
dAdo: 2′-deoxyadenosine
dGuo: 2′-deoxyguanosine
5FdU: 5-fluoro-2′-deoxyuridine
TFT: Trifluorothymidine
AZT: Azidothymidine
FLT: 2′-deoxy-3′-fluorothymidine
D4T: 2′,3′-dideoxythymidine
FMAU: 2′-deoxy-2′-fluoro-arabinofuranosyl-5-methyluracil
BvdU: 5-bromovinyl-2′-deoxyuridine
Urd: Uridine
IBD: Inflammatory bowel disease
